# Cyclic-AMP mediated drugs: differential or global reduction of eicosanoid synthesis in the isolated rat lung?

**DOI:** 10.1155/S0962935192000395

**Published:** 1992

**Authors:** Mark J. Post, Jan Dirk te Biesebeek, Johan Wemer, Hans H. van Rooij, Freek J. Zijlstra, Arijan J. Porsius

**Affiliations:** 1 Department of Pharmacology Erasmus University P.O. Box 1738 Rotterdam 3000 DR The Netherlands; 2 Department of Pharmacotherapy Faculty of Pharmacy P.O. Box 80082 University of Utrecht Utrecht 3508 TB The Netherlands; 3 Laboratory for Experimental Cardiology Department of Cardiology University Hospital Utrecht HPN E 02.562 P.O. Box 85500 Utrecht 3508 GA The Netherlands

## Abstract

In this study the question was addressed whether cAMP mediated drugs induce a differential reduction of branches of the arachidonic acid metabolism rather than a global reduction of eicosanoid synthesis. The isolated lungs of actively sensitized rats were employed to study prostaglandin and leukotriene release in the presence and absence of the cAMP mediated drugs theophylline, milrinone, sulmazole, isobutyl-methylxanthine and salbutamol. The release of eicosanoids as measured by RIA was predominantly basal and continuous, with a mild antigen induced stimulation only for TXB_2_ and the leukotrienes. All drugs reduced eicosanoid release globally. It is concluded that cAMP mediated drugs interfere with arachidonic acid metabolism at a site proximal to the branching into lipoxygenase and cyclo-oxygenase pathways.


Mediators of Inflammation, 1, 255–261 (1992)

**In this study the question was addressed whether cAMP mediated drugs induce a differential reduction of branches of the arachidonic acid metabolism rather than a global reduction of eicosanoid synthesis. The isolated lungs of actively sensitized rats were employed to study prostaglandin and leukotriene release in the presence and absence of the cAMP mediated drugs theophylline, milrinone, sulmazole, isobutyl-methylxanthine and salbutamol. The release of eicosanoids as measured by RIA was predominantly basal and continuous, with a mild antigen induced stimulation only for $TXB_2$ and the leukotrienes. All drugs reduced eicosanoid release globally. It is concluded that cAMP mediated drugs interfere with arachidonic acid metabolism at a site proximal to the branching into lipoxygenase and cyclo-oxygenase pathways.**

**Key words:** AA 861, cAMP, Leukotrienes, Lung, Milrinone, Prostaglandins

### Cyclic-AMP mediated drugs: differential or global reduction of eicosanoid synthesis in the isolated rat lung?

**Mark J. Post,*,CA Jan Dirk te Biesebeek, Johan Wemer,* Hans H. van Rooij, Freek J. Zijlstra[1] and Arijan J. Porsius**

Department of Pharmacotherapy, Faculty of Pharmacy, P.O. Box 80082, 3508 TB University of Utrecht, Utrecht, The Netherlands;
[1]Department of Pharmacology, Erasmus University, P.O. Box 1738, 3000 DR Rotterdam, The Netherlands
*Present address: Laboratory for Experimental Cardiology, Department of Cardiology, University Hospital Utrecht HPN E 02.562, P.O. Box 85500, 3508 GA Utrecht, The Netherlands

CA Corresponding Author

### Introduction

Drugs that reduce eicosanoid synthesis might be valuable in symptomatic and curative therapy of various inflammatory diseases. Such drugs include cAMP elevating compounds like the xanthine derivatives theophylline and IBMX, and $\beta$-sympaticomimetic drugs and inhibitors of cyclic nucleotide phosphodiesterase isoenzymes.[1,2] Milrinone and sulmazole are two recently developed cardiotonic and vasodilator drugs that belong to the latter category.[3,4] In previous experiments we compared the effects of milrinone, sulmazole and theophylline in the rat isolated lung. We showed that these drugs inhibited antigen induced bronchoconstriction, vasoconstriction and SRS-A release, leaving the release of the preformed mediators histamine and serotonin unaffected.[5] Rossing *et al.* also showed a bronchorelaxant effect of milrinone against a variety of agonists in trachea, lung parenchyma and pulmonary artery, that could be counteracted by indomethacin.[6] This led them to hypothesize that milrinone exerted a differential effect on arachidonic acid metabolism and either induced the synthesis of bronchorelaxing prostaglandins or reduced indomethacin enhanced production of bronchoconstrictive leukotrienes. In this study we addressed the question of whether cAMP mediated drugs induce a differential reduction of branches of the arachidonic acid metabolism rather than a global reduction of eicosanoid synthesis. We incorporated AA 861 in the study as a selective inhibitor of leukotriene synthesis[7,8] to check whether selective modulation of arachidonic acid metabolism could be shown in our model.

### Materials and Methods

*Sensitization:* Male Wistar rats, weighing 180–200 g, were actively sensitized. For this purpose, an emulsion was made of trinitrophenylovalbumin in aluminium phosphate, containing 50 $\mu g\ ml^{-1}$ TNP-OVA and 6 mg/ml $AlPO_4$. Of this solution, 2 ml was injected intraperitoneally. After 12–21 days the animals were used for experiments.

*Rat isolated and ventilated lungs with vascular perfusion:* Lungs were removed under pentobarbitone-Na anaesthesia (50 mg $kg^{-1}$ i.p.), suspended in a Plexiglas chamber and perfused through the pulmonary artery with Krebs–Ringer solution (37°C and gassed with 5% $CO_2$ in $O_2$) at a rate of 7.5 ml $min^{-1}$. The lungs were ventilated (25 $min^{-1}$) with a Braun piston pump by applying vacuum within the surrounding chamber, alternating from $-6.5$ cm $H_2O$ to $-1$ cm $H_2O$. A constant flow of $O_2$ with 5% $CO_2$ into the trachea imposed a positive pressure to the trachea, which was kept constant at 5.5 cm $H_2O$, by means of an adjustable

© 1992 Rapid Communications of Oxford Ltd water seal connected with a T-piece to the inflation tube. Thus, the end expiratory pressure was constant, while the end inspiratory pressure rose as a function of airway diameter. The area under the end inspiratory pressure curve was measured and expressed as a percentage of the area under the (horizontal) curve representing the maximally attainable end inspiratory pressure. Pulmonary artery pressure and tracheal pressure were measured by means of an Ailtech (MEDIO-E) and an Elema-Schönander transducer, respectively. Each pair of lungs was allowed to equilibrate for 15 min. After antigen challenge, perfusate was continuously collected for a period of 7 min and analysed for mediators. In one series of experiments, designed to study the influence of antigen challenge on eicosanoid release, a triplet of 5 min perfusate samples was collected: one before antigen challenge, a second from 1 to 6 min after antigen challenge and a third from 10 to 15 min after challenge.

*Antigen challenge and drug treatment:* Lungs were challenged with a bolus injection of 0.2 ml (50 $\mu g\ ml^{-1}$) undiluted TNP-OVA solution, into the pulmonary artery. This proved to be a supramaximal dose in previous studies.

All drugs were added to the perfusion fluid during the entire course of the experiments starting at least 15 min before antigen challenge. The drugs were either dissolved in the perfusion fluid or added to the infusion fluid by means of a constant infusion just prior to the entrance into the pulmonary artery.

*Determination of mediators:* Histamine was measured fluorometrically after pre-column derivatization with *o*-phthalaldehyde and subsequent separation by means of HPLC. All eicosanoids were measured by RIA. For this purpose, perfusate was collected in 1 min fractions, from 1 to 7 min after antigen challenge. Each fraction was applied to a Sep-Pak $C_{18}$ cartridge. The cartridges had been prewashed with ethanol, water and, finally, 0.5% EDTA in water (pH = 5.5). Prior to elution with 4 ml ethanol, the cartridges were flushed with 5 ml water. [$^3$H]-$PGA_2$ was used as an internal standard to determine recovery. Samples of 50 or 100 $\mu l$ were dried and dissolved in RIA buffer. Detection ranges were: 5-HETE (0–500 ng), 12-HETE (0–1000 ng), 15-HETE (0–250 ng), $PGE_2$ (0–1000 ng), 6k-$PGF_{1\alpha}$ (0–250 ng), $PGF_{2\alpha}$ (0–500 ng), $TXB_2$ (0–250 ng), $LTB_4$ (0–500 ng) and peptidoleukotrienes (0–500 ng). The detection limits for the prostaglandins, peptidoleukotrienes and HETEs were 2, 5 and 10 pg respectively.

Cross reactivities for radioimmunoassays of LTs, HETEs and prostaglandins (% at 50% $B/B_0$), were less than 1%, except for anti-5-HETE with $LTB_4$ (4%), anti-$PGF_{2\alpha}$ with 6k-$PGF_{1\alpha}$ (2.2%) and anti-$PGF_{2\alpha}$ with $PGE_2$ (1.3%). The peptidoleukotrienes assay discriminates rather poorly between $LTC_4$, $LTD_4$, $LTE_4$ or $LTF_4$, cross-reactivities with these LTs being 55%, 100%, 51% and 67% respectively.

*Drugs and materials:* Antisera and standards for RIAs were obtained from Advanced Magnetics Inc., (Cambridge, MA, USA) and $^3$H-labelled antigen from the Radiochemical Centre, (Amersham, UK). Ovalbumin, grade V, was purchased from Sigma. Trinitrobenzenesulphonic acid was obtained from Baker Chem. (Deventer, The Netherlands). Sulmazole and milrinone were gifts from Dr Karl Thomae GmbH (Biberach, Germany) and Sterling-Winthrop (USA), respectively. AA 861 (2–12-hydroxy-dodeca-5,10-dinyl)-3,5,6-trimethyl-1,4-benzoquinone) was supplied by Takeda Chemical Industries Ltd (Osaka, Japan). Theophylline, salbutamol and isobutylmethylxanthine (IBMX) were all purchased from Sigma. Sulmazole, theophylline, salbutamol and IBMX were dissolved in the perfusion medium, while milrinone and AA 861 were administered with an infusion to the perfusion medium. For this purpose, milrinone was dissolved in Krebs acidified with hydrochloric acid to a pH of 4.5, not affecting the pH of the perfusion fluid. AA 861 was dissolved in DMSO (final concentration 0.3%). All constituents of the perfusion fluid, ethanol and *o*-phthalaldehyde were obtained from Merck, and were of analytical grade. Sep-Pak $C_{18}$ cartridges were purchased from Millipore Co.

*Data analysis:* In the text and figures, data represent the arithmetic means $\pm$SE. Mediator release was expressed as total amount present in the perfusate collected from 1 to 7 min after challenge (yielding 45 ml perfusate). Three control groups were incorporated in this study, one matched with the theophylline 10 and 100 $\mu M$, milrinone and sulmazole treated groups, a second matched with the theophylline 1 mM, IBMX and salbutamol group and a third DMSO 0.3% control group. In the second control group, time dependency of mediator release was studied by collecting and analysing 1 min fractions separately, again from 1 to 7 min after challenge. The sum of these fractions were used for comparison with the drug treated groups (theophylline 1 mM, IBMX, salbutamol). Differences in mean bronchoconstriction and mediator release were evaluated by means of a one-way analysis of variance on raw data, comparing the absolute figures from treated groups with their respective control groups. If the F-test showed significant differences between groups ($p < 0.05$), differences in means were evaluated with Duncan's multiple range test and judged to be significant if $p < 0.05$.

### Results

*Time dependency of mediator release after antigen challenge:* (a) Preformed mediators. Antigen challenge induced a rapid and substantial release of histamine up to 857 ± 105 ng min$^{-1}$, $n = 7$ (Fig. 1a). A total of approximately 2 000 ng histamine was released by each pair of lungs (weighing approximately 1 g). Within 1 min after challenge, maximal concentrations of histamine were measured. After 1 min histamine release gradually subsided, reaching basal release (227 ± 43 ng min$^{-1}$, $n = 7$) in 7 min. (b) Eicosanoids. The mono-HETEs, 5-HETE, 12-HETE and 15-HETE, were synthesized and released by the rat isolated lung. Of these, the release of 12-HETE was the most predominant (Fig. 1b). 15-HETE and 5-HETE were released in small amounts only (±500 pg min$^{-1}$) (Fig. 1c).

$PGI_2$, measured as 6k-$PGF_2$ was the predominant bronchorelaxant prostaglandin. Neither $PGI_2$, nor $PGE_2$ showed a clear release pattern after antigen stimulation (Fig. 1d).

The bronchoconstrictive prostaglandin $PGF_{2\alpha}$ and the stabile metabolite of thromboxane $A_2$, $TXB_2$, were released in equal amounts (±1 000 pg min$^{-1}$) (Fig. 1e). The release of $TXB_2$ was stimulated significantly at 2 min after antigen challenge, declining thereafter.

$LTB_4$ and the peptidoleukotrienes, were released in relatively small amounts by the rat lung (Fig. 1f). $LTB_4$ showed a clear release pattern after antigen stimulation reaching its maximum at $t = 3$ min.

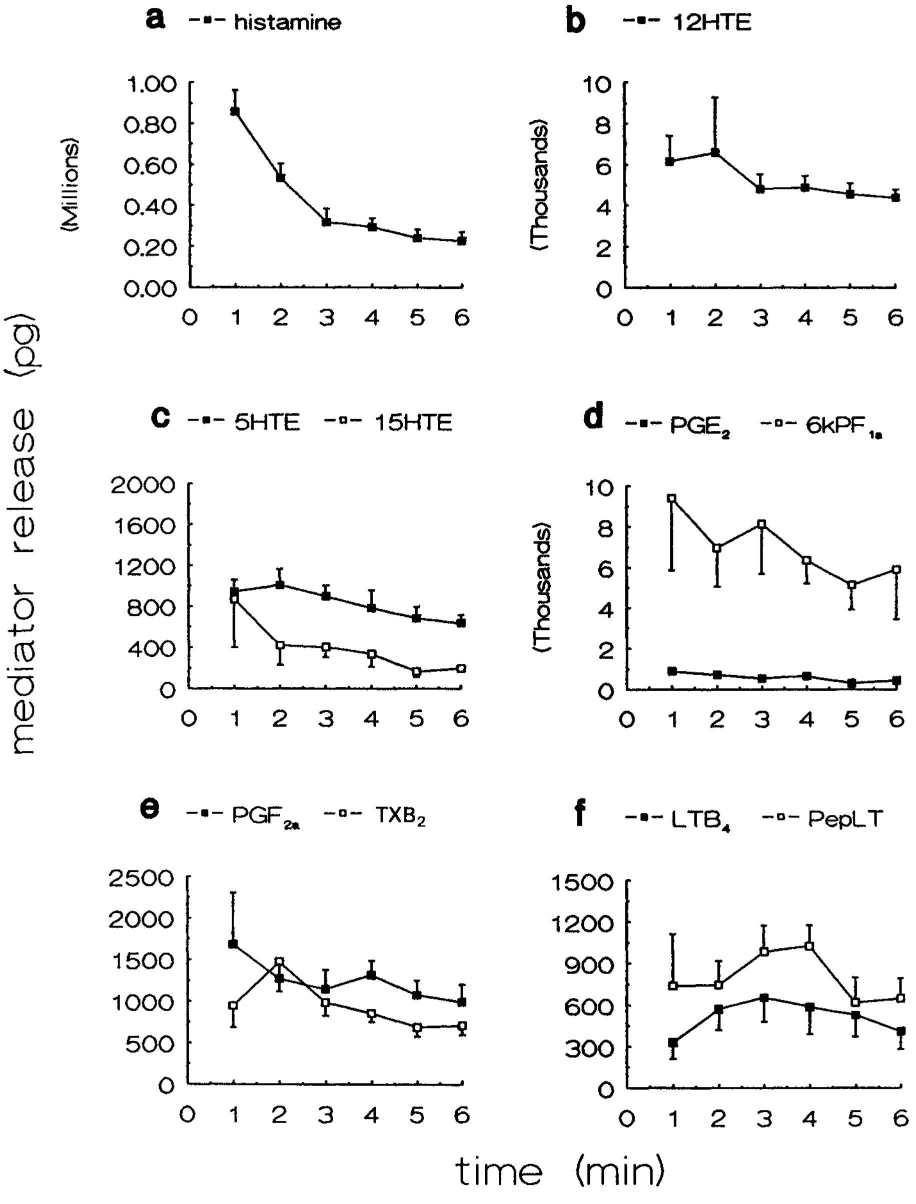

FIG. 1. Release of mediators at various times after antigen challenge. Release of histamine (a), 12-HETE (b), 5- and 15-HETE (c), $PGE_2$ and 6k-$PGF_{1\alpha}$ (d), $PGF_{2\alpha}$ and $TXB_2$ (e), and the peptidoleukotrienes and $LTB_4$ (f). Antigen challenge was given at t = 0. The data represent the means of six experiments, the bars indicate the SE. Notice the different scales on the ordinates of each panel.

Peptidoleukotriene release was also stimulated, with a maximal release at $t = 4$ min.

In four separate experiments, we attempted to establish a release pattern for the peptidoleukotrienes, 12-HETE and $TXB_2$, by collecting a triplet of 5 min perfusate fractions. The results are shown in Fig. 2. A stimulatory release pattern could be observed for the peptidoleukotrienes and $TXB_2$. Antigen challenge in the rat lung did not stimulate the release of 12-HETE and $6k\text{-}PGF_{1\alpha}$.

For all eicosanoids, antigen-induced synthesis was low and in most instances undistinguishable from basal release (Figs 1 and 2). In the experiments where modulation of mediator release by drugs was studied, total, i.e., basal and antigen induced release, was measured.

*Modulation of mono-HETE production by the drugs:* The mono-HETEs were quite uniformly affected by theophylline, milrinone and sulmazole (Table 3). These drugs reduced mono-HETE production at high concentrations only, with the exception of 15-HETE, the production and release of which seemed to be resistant to sulmazole. Salbutamol, IBMX and AA 861 reduced 5-HETE, but not 12-HETE production.

*Bronchoconstriction and total mediator release by antigen challenged lungs:* Table 1 lists the results of antigen induced bronchoconstriction and total mediator release in the three control groups ($n = 6$). As mentioned, we performed the experiments described here in different seasonal periods and therefore had two different control groups and one vehicle control group. No significant differences in mediator release between these groups were observed, except for 5-HETE and $LTB_4$. In control group 2, $PGE_2$ measurement failed.

*Modulation of antigen induced bronchoconstriction and total histamine release:* In Table 2 the dose dependent inhibition of antigen induced bronchoconstriction by theophylline, milrinone, sulmazole, IBMX and salbutamol is depicted. AA 861 at a concentration of 5 $\mu$M did not affect antigen induced bronchoconstriction. Histamine release was rather insensitive to the action of various drugs. Only sulmazole at a concentration of 100 $\mu$M, reduced the release of this autocoid (Table 2). Theophylline 1 mM was not

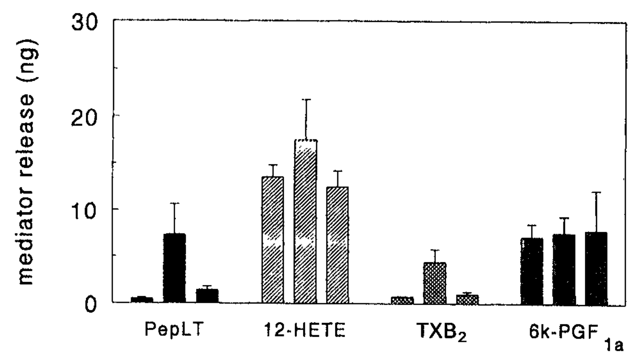

FIG. 2. Release patterns of peptidoleukotrienes (filled bars), 12-HETE (12-HETE, coarse right-hatched bars), $TXB_2$ (cross-hatched bars) and $6k\text{-}PGF_{1\alpha}$ ($6kPF_{1\alpha}$, fine right-hatched bars) when sensitized lungs are challenged with antigen. Perfusate fractions were collected for 5 min before antigen challenge, from 1 to 6 min and from 10 to 15 min after antigen challenge, represented in each cluster of bars by the first, second and third bar, respectively. The data indicate the means ±SE of four experiments.

**Table 1.** Bronchoconstriction and mediator release in control groups

| | Control 1 | Control 2 | Control 3 (DMSO) |
|---|---|---|---|
| AUC (%) | 32.0 ± 3.8 | 28.9 ± 4.8 | 30.7 ± 4.8 |
| Histamine (ng) | 1774 ± 178 | 2399 ± 362 | 2294 ± 437 |
| 5-HETE (ng) | 3.0 ± 0.4 | 4.7 ± 0.1* | 3.6 ± 0.6 |
| 12-HETE (ng) | 26.4 ± 2.1 | 25.6 ± 1.4 | 26.9 ± 6.2 |
| 15-HETE (ng) | 1.2 ± 0.1 | 1.8 ± 0.3 | 1.5 ± 0.4 |
| $PGE_2$ (ng) | 22.6 ± 5.4 | ND | 20.8 ± 2.1 |
| $6k\text{-}PGF_{1\alpha}$ (ng) | 60 ± 18 | 40 ± 11 | 25.1 ± 2.9 |
| $PGF_{2\alpha}$ (ng) | 7.0 ± 2.0 | 7.5 ± 1.0 | 5.9 ± 0.9 |
| $TXB_2$ (ng) | 5.4 ± 0.6 | 5.7 ± 0.9 | 4.6 ± 0.9 |
| $LTB_4$ (ng) | 12.1 ± 1.9 | 3.1 ± 0.8* | 11.6 ± 1.4 |
| PLTs (ng) | 3.5 ± 0.2 | 4.8 ± 1.0 | 2.9 ± 0.4 |

Control groups were studied at different periods. Although the lungs were challenged with antigen, total (antigen induced and basal) mediator release is depicted (means ±SE). The asterisks indicate values that are significantly different from the values in the other two groups ($p < 0.05$, $n = 6$). ND: not determined. AUC: area under curve, bronchoconstriction; PLTs: peptidoleukotrienes.

**Table 2.** Modulation of total histamine release and antigen induced bronchoconstriction

| | AUC (%) | HR (%) |
|---|---|---|
| **Theophylline** | | |
| 0.01 mM | 24 ± 13* | −5 ± 25 |
| 0.1 mM | 29 ± 12* | 27 ± 19 |
| 1 mM | 73 ± 6** | ND |
| **Milrinone** | | |
| 0.01 mM | 25 ± 11* | −2 ± 16 |
| 0.1 mM | 50 ± 8** | 21 ± 18 |
| **Sulmazole** | | |
| 0.01 mM | 31 ± 10* | 31 ± 10 |
| 0.1 mM | 72 ± 5* | 33 ± 10* |
| **IBMX** | | |
| 10 $\mu$M | 54 ± 8** | 12 ± 15 |
| **Salbutamol** | | |
| 1 $\mu$M | 11 ± 18 | 8 ± 14 |
| 10 $\mu$M | 36 ± 10* | 15 ± 16 |
| **AA 861** | | |
| 5 $\mu$M | 13 ± 11 | 21 ± 10 |

Data are expressed as percentage inhibition of control data of bronchoconstriction and histamine release as given in Table 1, and represent means ±SEM, $n = 6$ for all (the SEM is calculated from the variances of both the control and the treated values). The asterisks indicate significance of difference with controls at $p < 0.05$ or, if double, at $p < 0.01$. AUC: area under curve; HR: histamine release; ND: not determined.

**Table 3.** Modulation of total eicosanoid release

| | 5-HETE | 12-HETE | 15-HETE | $PGE_2$ | $6PGF_{1\alpha}$ | $PGF_{2\alpha}$ | $TXB_2$ | $LTB_4$ | pepLT |
|---|---|---|---|---|---|---|---|---|---|
| **Theophylline** | | | | | | | | | |
| 0.01 mM | 0 | 7 ± 12 | 12 ± 13 | 79 ± 8* | 61 ± 16* | 27 ± 24 | 1 ± 17 | 24 ± 13 | 0 |
| 0.1 mM | 7 ± 20 | 3 ± 13 | 29 ± 7* | 86 ± 5* | 80 ± 6* | 49 ± 15* | 22 ± 17 | 43 ± 13* | 0 |
| 1 mM | 78 ± 3* | 40 ± 12* | 83 ± 6* | ND | 75 ± 7* | 62 ± 6* | 74 ± 5* | 82 ± 8* | 87 ± 4* |
| **Milrinone** | | | | | | | | | |
| 0.01 mM | 0 | 23 ± 11 | 17 ± 13 | 82 ± 7* | 80 ± 7* | 47 ± 15* | 34 ± 11* | 29 ± 19 | 0 |
| 0.1 mM | 31 ± 11* | 29 ± 8* | 37 ± 12* | 86 ± 8* | 80 ± 6* | 54 ± 14* | 76 ± 5* | 66 ± 7* | 35 ± 5* |
| **Sulmazole** | | | | | | | | | |
| 0.01 mM | 28 ± 14 | 6 ± 10 | 26 ± 9 | 56 ± 22* | 74 ± 10* | 51 ± 16* | 35 ± 8* | 44 ± 12* | 22 ± 12 |
| 0.1 mM | 52 ± 7* | 53 ± 5* | 26 ± 15 | 95 ± 4* | 87 ± 5* | 82 ± 5* | 89 ± 3* | 83 ± 4* | 51 ± 9* |
| **Salbutamol** | | | | | | | | | |
| 1 μM | 66 ± 2* | 36 ± 13 | ND | ND | 58 ± 13* | 67 ± 7* | 64 ± 10* | 32 ± 20 | 78 ± 6* |
| 10 μM | 57 ± 5* | ND | ND | ND | 66 ± 10* | 66 ± 5* | 56 ± 13* | 20 ± 33 | 73 ± 9* |
| **IBMX** | | | | | | | | | |
| 10 μM | 76 ± 2* | 6 ± 21 | ND | ND | 81 ± 5* | 67 ± 5* | 59 ± 8* | 48 ± 19* | 70 ± 6* |
| **AA 861** | | | | | | | | | |
| 5 μM | 65 ± 9* | 4 ± 25 | 65 ± 10* | 90 ± 3** | 30 ± 19 | 43 ± 12* | 20 ± 21 | 99 ± 2** | 73 ± 3** |

Data are expressed as percentage inhibition of control data of bronchoconstriction and mediator release as given in Table 1 (theophylline 0.01 and 0.1 mM, milrinone and sulmazole, control 1; theophylline 1 mM, salbutamol and IBMX, control 2; AA 861, control 3) and represent means ±SE (the SE is calculated from the variances of control and treated values). The asterisks indicate significant differences with controls at $p < 0.05$ or, if double, at $p < 0.01$. One-way analysis of variance was performed on raw data, not on percentages. '0' is entered whenever the calculated percentage of inhibition was negative, without being significantly different from zero. In no case was a potentiation of arachidonic acid metabolism observed. ND: not determined.

evaluated, since the aminophylline used for these high concentrations interfered with the fluorometric detection of histamine.

*Modulation of bronchorelaxant prostaglandins:* The production of the bronchorelaxant prostaglandin 6k-$PGF_{1\alpha}$ was inhibited by five of the six drugs tested. AA 861 had no effect (Table 3). For sulmazole, salbutamol and theophylline obvious dose dependency was observed, while milrinone apparently had reached its maximal effect at 0.01 mM. $PGE_2$ showed a pattern similar to 6k-$PGF_{1\alpha}$. Since $PGE_2$ data fail in the second control group, the effect of theophylline 1 mM, IBMX, salbutamol could not be calculated. However, in absolute figures, the amounts of $PGE_2$ were comparable to the other treated groups, ranging from 1.7 to 5.3 ng, as opposed to 22.6 ng in control group 1 (Table 2). Surprisingly, $PGE_2$ production was strongly reduced in the AA 861 treated group.

*Modulation of bronchoconstrictive prostaglandins:* Production of $PGF_{2\alpha}$ and $TXB_2$ was effectively reduced by all cAMP mediated drugs (Table 3). For theophylline, milrinone and sulmazole a dose dependency was observed. Increasing the dose of salbutamol from 1 to 10 μM had no additional effect on leukotriene production. AA 861 caused a moderate reduction of $PGF_{2\alpha}$, leaving $TXB_2$ synthesis unaffected.

*Modulation of leukotriene production:* Synthesis of $LTB_4$ and the peptidoleukotrienes were reduced by theophylline and milrinone at high concentrations. Sulmazole and IBMX already at 0.01 mM reduced $LTB_4$ production significantly (Table 3). AA 861 almost abolished $LTB_4$ and peptidoleukotriene synthesis. In contrast, salbutamol had no affect on $LTB_4$ synthesis.

### Discussion

In this paper we confirmed and extended earlier observations on the reduction of synthesis and release of peptidoleukotrienes by milrinone, sulmazole and theophylline in the rat isolated lung.[5] Rossing and coworkers[6] postulated a selective induction of bronchorelaxant prostaglandins by milrinone to explain the inhibition of milrinone induced bronchorelaxation by indomethacin. However, our data show that these and other supposedly cAMP mediated drugs reduce arachidonic acid metabolism in a global rather than a differential manner.

Global reduction of arachidonic acid metabolism by cAMP mediated drugs suggests interference with an early stage of arachidonic acid metabolism prior to separation into the lipoxygenase and cyclooxygenase pathway. This might be explained by an evaluation of intracellular cAMP reducing the release of arachidonic acid from the phospholipid pool. It has been shown for instance that beta agonists, by stimulation of adenylate cyclase, reduce phospholipase $A_2$ activity and the subsequent release of arachidonic acid metabolites.[9,10]

In accordance with other reports, histamine release was not affected by the drugs tested.[5,11,12]

Apparently, production of histamine release is less susceptible to elevation of cAMP than eicosanoid production.[9,13]

For theophylline, milrinone and sulmazole, mechanisms of action other than cAMP elevation have been proposed. Antagonism of adenosine receptors is the most strongly advocated alternative. On the basis of previous experiments we argued against adenosine antagonism as a likely mechanism of action for milrinone and sulmazole in the concentrations used in this study.[14]

Milrinone is a selective inhibitor of the phosphodiesterase enzyme complex III,[3] which hydrolyses mainly cAMP. Whether this selectivity still holds in the rat lung for concentrations of 0.01 to 0.1 mM is uncertain. In view of the similarity of the pharmacological profiles of theophylline, IBMX, sulmazole and theophylline it is likely that nonselective inhibition of the cyclic nucleotide phosphodiesterase isozyme complex is responsible for the observed effects.

Using RIA, a wide spectrum of eicosanoids could be measured in the perfusate of the isolated rat lungs, comprising major representatives of the two most important pathways of arachidonic acid metabolism. Of the mono-HETEs, 12-HETE was the most predominant, whereas only small amounts of 15- and 5-HETE were produced. The same holds for guinea-pigs, whereas in man, 15-HETE production is more pronounced.[15] The quantities of arachidonic acid metabolites measured in our experiments, are largely correlated with those found in rat mast cells, human lung parenchyma and the guinea-pig isolated lung.[15-17] $TXB_2$ and leukotrienes however seem to be more prominent in the guinea-pig than in the rat. The production of histamine, $TXB_2$, $LTB_4$ and the peptidoleukotrienes was stimulated by antigen, as was also shown by Turner and Dollery (1988), using the isolated guinea-pig lung.[18] In contrast, no clear stimulation of 6k-$PGF_{1\alpha}$, $PGF_{2\alpha}$, $PGE_2$ and the mono-HETEs was observed in our study. Thus, it is likely that in this model the release of mono-HETEs and prostaglandins is mainly basal. Whether this reflects a constant low-level stimulation of the isolated lung is unknown. The reduction of eicosanoid release by drugs shows the reversibility of eicosanoid release in this model, ruling out leakage of eicosanoids from damaged cells.

AA 861 was introduced in this study to validate the study design. A partial selectivity in reducing arachidonic acid metabolism could indeed be demonstrated. As expected, AA 861 caused an almost complete cessation of leukotriene production in our experiments, while thromboxane and $PGI_2$ remained unaffected. However, the production of 15-HETE, $PGF_{2\alpha}$ and $PGE_2$ was reduced as well. In contrast, at AA 861 concentrations up to 10 $\mu$M, no inhibition of human platelet cyclooxygenase or $PGE_2$ production by mouse myocardial cells was observed in other studies.[8,19] Recently it became clear that AA 861 was not selective under all conditions and in all animal strains.[20,21] In human mast cells from the skin and the lung for instance, AA 861 potently inhibited anti-IgE induced $PGD_2$ release.[22] The selectivity of this agent obviously needs further documentation.

Antigen induced production of leukotrienes and other eicosanoids is probably not directly responsible for early antigen induced bronchoconstriction. The drugs tested in this study reduced bronchoconstriction at concentrations that were inadequate for inhibition of leukotriene production. A similar observation has been made for forskolin.[23] In view of these data, our finding that AA 861 had no effect on bronchoconstriction, while almost abolishing leukotriene production, supports the prevailing notion that leukotrienes have little significance in early anaphylactic bronchoconstriction in the rat.[24,25]

In conclusion, all cAMP mediated drugs reduced antigen induced bronchoconstriction and the production of eicosanoids in isolated rat lungs. Arachidonic acid metabolism is impeded at a site proximal to the separation into lipoxygenase and cyclooxygenase pathways. Whether differences in this respect exist between phosphodiesterase inhibitors and stimulators of adenylate cyclase, remains to be established. The results with AA 861 show that immediate antigen induced bronchoconstriction is not mediated by leukotrienes. Finally, we suggest that AA 861 might not in all conditions be a selective 5-lipoxygenase inhibitor.

### References

1. Peachell PT, MacGlashan JDW, Lichtenstein LM, Schleimer R. Regulation of human basophil and lung mast cell function by cyclic adenosine monophosphate. *J Immunol* 1988; **140**: 571–579.
2. Warner JA, MacGlashan JDW, Peters SP, Kagey-Sobotka A, Lichtenstein LM. The pharmacologic modulation of mediator release from human basophils. *J Allergy Clin Immunol* 1988; **82**: 432–438.
3. Weishaar RE, Burrows SD, Kobylarz DC, Quade MM, Evans DB. Multiple molecular forms of cyclic nucleotide phosphodiesterase in cardiac and smooth muscle and in platelets. *Biochem Pharmacol* 1986; **35**: 787–800.
4. Beavo JA, Reifsnyder DH. Primary sequence of cyclic nucleotide phosphodiesterase isozymes and the design of selective inhibitors. *Trends Pharmacol Sci* 1990; **11**: 150–155.
5. Post MJ, te Biesebeek JD, van Rooij HH, Werner J, Porsius AJ. Anti-allergic effects of milrinone and sulmazole in isolated rat lungs in comparison with theophylline. *Int Arch Allergy Appl Immunol* 1989; **89**: 1–6.
6. Rossing TH, Drazen JM. Effects of milrinone on contractile responses of guinea pig trachea, lung parenchyma and pulmonary artery. *J Pharmacol Exp Ther* 1986; **238**: 874–879.
7. Saito H, Hirai A, Tamura Y, Yoshida S. The 5-lipoxygenase products can modulate the synthesis of platelet activating factor (alkyl-acetyl GPC) in Ca-ionophore A23187 stimulated rat peritoneal mast cells. *Prost Leukotr Med* 1985; **18**: 271–286.
8. Ikeda U, Toyo-oka T, Arisaka H, Hosoda S. Stimulated synthesis of prostaglandin E2 or leukotriene C4 from myocardial cells is not a cause but a result of their injury under hypoxia. *J Mol Cell Cardiol* 1987; **19**: 523–527.
9. Undem BJ, Peachell PT, Lichtenstein LM. Isoproterenol-induced inhibition of immunoglobulin E mediated release of histamine and arachidonic acid metabolites from the human lung mast cell. *J Pharmacol Exp Ther* 1988; **247**: 209–217.

10. Wichert van P, Meyer W. *In vivo* inhibition of phospholipase A2 in rat lung by an $\beta$-adrenergic agonist. *Agents & Actions* 1983; **13**: 233–234.
11. Sydbom A, Fredholm BB. On the mechanism by which theophylline inhibits histamine release from rat mast cells. *Acta Physiol Scand* 1982; **114**: 243–251.
12. Church MK. Modulation of mast cell mediator secretion by drugs used in the treatment of allergic diseases. In: Holgate ST, ed. *Mast cells, mediators and disease.* London: Kluwer Academic Publishers, 1988; 259–279.
13. Orange RP, Kaliner MA, Laraia PJ, Austen KF. Immunological release of histamine and slow reactive substance of anaphylaxis from human lung. II. Influence of cellular levels of cyclic AMP. *Fed Proc* 1971; **6**: 1725–1729.
14. Post MJ, te-Biesebeek JD, Wemer J, van Rooij HH, Porsius AJ. Effects of milrinone, sulmazole and theophylline on adenosine enhancement of antigen-induced bronchoconstriction and mediator release in rat isolated lungs. *Pulm Pharmacol* 1991; **4**: 239–246.
15. Grupe R. Lipoxygenasekatalysierter arachidonsäure-metabolismus und auswirkungen seiner inhibition auf anaphylaktische reaktionen. *Pharmazie* 1986; **41**: 7–22.
16. Schulman ES, Newball HN, Demers LM, Fitzpatrick FA, Adkinson JNF. Anaphylactic release of thromboxane $A_2$, prostaglandin $D_2$ and prostacyclin from human lung parenchyma. *Am Rev Respir Dis* 1982; **124**: 402–406.
17. Hanley SP. Prostaglandins and the lung. *Lung* 1986; **164**: 65–77.
18. Turner NC, Dollery CT. Release of arachidonic acid metabolites and histamine from sensitized guinea pig lung following antigen challenge. *Br J Pharmacol* 1988; **93**: 751–758.
19. Mita H, Yui Y, Shida T. Effect of AA 861, a 5-lipoxygenase inhibitor, on leukotriene synthesis in human polymorphonuclear leukocytes and on cyclooxygenase and 12-lipoxygenase activities in human platelets. *Allergy* 1986; **41**: 493–498.
20. Yamamura H, Taira M, Negi H, Nanbu F, Kohno S, Ohata K. Effect of AA 861, a selective 5-lipoxygenase inhibitor, on models of allergy in several species. *Jap J Pharmacol* 1988; **47**: 261–271.
21. Nakadate T, Yamamoto S, Aizu E, Kato R. Inhibition of mouse 12-lipoxygenase by 2,3,4-trimethyl-6-2,1,2-hydroxy-5,10-dodecadiynyl)-1,4-benzoquinone (AA 861). *J Pharmac Pharmacol* 1985; **37**: 71–73.
22. Cohan V, McKenzie-White J, Triggiani M, Massey W, Kagey-Sobotka A, Lichtenstein LM. Heterogeneity of human mast cells and basophils. Effects of a putative 5-lipoxygenase inhibitor. *Biochem Pharmacol* 1989; **38**: 4455–4459.
23. Kreutner W, Chapman RW, Gulbenkian A, Tozzi S. Bronchodilator and anti-allergic activity of forskolin. *Eur J Pharmacol* 1985; **111**: 1–8.
24. Dahlbäck M, Bergstrand H, Sörenby L. Bronchial anaphylaxis in actively sensitized Sprague Dawley rats: Studies on mediators involved. *Acta Pharmac Tox* 1984; **55**: 6–17.
25. Brunet G, Piechuta H, Hamel R, Holme G, Ford-Hutchinson AW. Respiratory responses to leukotrienes and biogenic amines in normal and hyperreactive rats. *J Immunol* 1983; **131**: 434–438.

**Received 29 April 1992; accepted in revised form 11 June 1992**
